# Posterior percutaneous endoscopic lumbar discectomy combined with the vertical anchoring technique for lumbar disc herniation with distant upward migration

**DOI:** 10.1186/s13018-019-1519-9

**Published:** 2019-12-27

**Authors:** Yu Xia, Qiongyue Zhang, Xiang Gao, Keran Wang, Xun Zhang, Yu Du, Liang Chen

**Affiliations:** Department of Orthopaedics, The Second Affiliated Hospital of Chongqing Medical University, No. 76 Linjiang Road, Yuzhong District, Chongqing, 400010 China

**Keywords:** Posterior percutaneous endoscopic lumbar discectomy, Lumbar disc herniation, Distant upward migration, Vertical anchoring technique

## Abstract

**Background:**

Posterior percutaneous endoscopic lumbar discectomy (PELD) has become a preferred procedure for the treatment of simple lumbar disc herniation (LDH) but has rarely been reported for distant upward migration. The purpose of this research was to investigate the feasibility, safety, clinical efficacy and technical points of posterior PELD combined with the vertical anchoring technique (VAT) for the treatment of LDH with distant upward migration.

**Methods:**

Thirteen patients with distant upward migrated LDH who underwent posterior PELD combined with the VAT from March 2016 to May 2018 were selected. Among these cases, the herniated disc was located at L3/4 in 2 patients, L4/5 in 9 patients and L5/S1 in 2 patients. The operative time, length of hospital stay and postoperative complications were recorded. The visual analogue score (VAS), Oswestry Disability Index (ODI), Japanese Orthopaedic Association (JOA) scores and modified MacNab criteria were used to assess surgical efficacy.

**Results:**

All 13 patients underwent successful surgery. We compared the VAS, ODI and JOA scores before and after surgery. The differences were statistically significant (*P* < 0.05). According to the modified MacNab criteria, 10 patients were assessed as “excellent”, 2 patients were assessed as “good” and 1 patient was assessed as “fair” at the last follow-up. The rate of satisfactory outcomes was 92.3%.

**Conclusion:**

Posterior PELD combined with the VAT is a safe and feasible procedure for the treatment of LDH with distant upward migration and represents a new approach for this type of surgery.

## Introduction

**Fig. 1:**
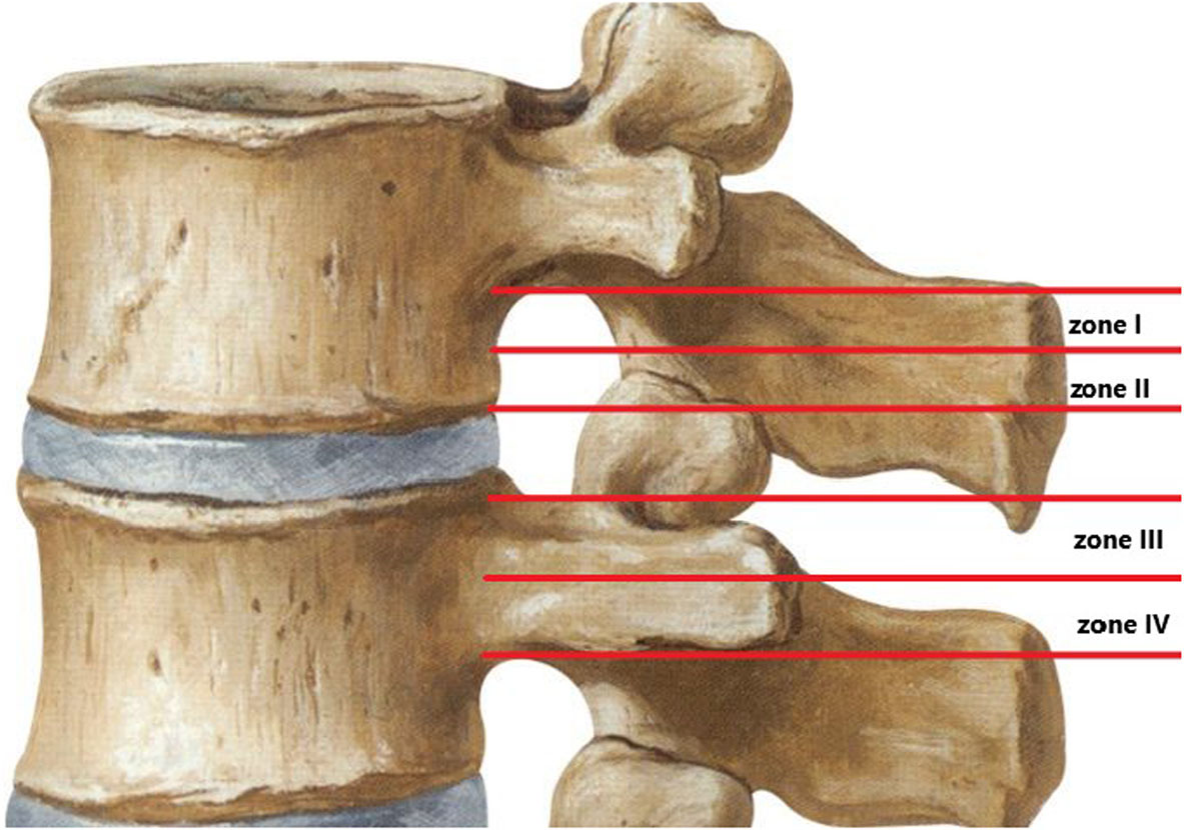
Four regions of the lumbar spine, as divided by Lee et al.: zone 1, the far-upward zone, within 3 mm of the lower edge of the upper pedicle; zone 2, the near-upward zone, between the lower edge of the upper vertebral body and 3 mm from the lower edge of the upper pedicle; zone 3, the near-downward zone, between the upper edge of the lower vertebral body and the midpoint level of the lower pedicle; and zone 4, the far-downward zone, between the midpoint of the lower pedicle and the lower edge of the lower pedicle

Posterior percutaneous endoscopic lumbar discectomy (PELD) is an emerging technique for the treatment of lumbar disc herniation (LDH), which has several advantages, including minimal trauma, a short operative time and a quick postoperative recovery. PELD has become a preferred procedure for the treatment of simple LDH but has rarely been reported for distant upward migration, which is a rare type of LDH [1–3]. Lee et al. divided the sagittal plane of the lumbar spine into 4 regions (Fig. 1) [4]. We further defined herniated discs in or above zone 1 as distant upward migration. Lee et al. believed that the intervertebral foramen approach for PELD is not suitable for the treatment of distant upward migration and thus suggested using open surgery. Choi et al. also analysed failed cases of endoscopic lumbar surgery and asserted that open surgery may be a safer and more effective option for this type of LDH [4–6].

However, open surgery has shortcomings, including considerable trauma and a slow postoperative recovery, and significantly impacts the stability of the spine, substantially increasing the risk of degeneration in adjacent segments [7, 8]. Developing a minimally invasive technique to solve this problem has always been our goal. The traditional intervertebral foramen approach often requires removal of most of the facet joints to excise a distant upward migrated disc. This method can reduce the risk of residual herniated discs. However, damage to the stability of the facet joints compromises the original intention of minimally invasive surgery. The interlaminar approach can be used to remove L5/S1 herniated discs in some patients but is suitable only for selected patients because of the limited size of the interlaminar gap [9, 10]. Based on pars interarticularis fenestration introduced by Di Lorenzo et al. in 1998 [11], we found that safely and completely removing distant upwardly migrated disc tissue via an osseous channel established by the posterior approach is theoretically feasible. From March 2016 to May 2018, we used posterior PELD combined with the vertical anchoring technique (VAT) to treat 13 patients with LDH with distant upward migration. The technical points and short-term effects are summarized below.

## Materials and methods

### General data

Among the 13 patients, 8 were males, and 5 were females, with a mean age of 53.4 ± 13.4 years (range 31–74 years). The herniated disc was located at L3/4 in 2 patients, L4/5 in 9 patients and L5/S1 in 2 patients (Table 1). The inclusion criteria were as follows: (1) main patient complaint, such as radiation pain or numbness in the unilateral lower extremity with or without low back pain, related to nerve root compression; (2) complete preoperative imaging data; (3) sagittal T2-weighted images showing that the herniated disc was located in or above zone 1 and compressed the corresponding nerve root, reflecting an imaging examination consistent with the symptoms and signs; and (4) symptoms that were not improved or worsened after more than 6 weeks of regular conservative treatment. The exclusion criteria were as follows: (1) concomitant lumbar spinal stenosis; (2) concomitant foraminal stenosis; (3) lumbar instability, spondylolisthesis, deformity, fracture, or tumour; and (4) an inability to tolerate surgery due to other severe concurrent diseases.

**Table 1:** Patients’ demographic characteristics

Gender		Age (years)	Treatment level		
Male	Female	(Mean ± SD)	L3/4	L4/5	L5/S1
8	5	53.4 ± 13	2	9	2

### Surgical procedure

#### Surgical instruments

All surgical instruments were manufactured by Spinendos, Inc., Germany, and included an endoscope, endoscopic sheaths, 3-mm high-speed grinding drills, nucleus pulposus clamp, laminectomy rongeurs, a trepan and a bipolar radiofrequency (RF) device.

#### Patient positioning

**Fig. 2:**
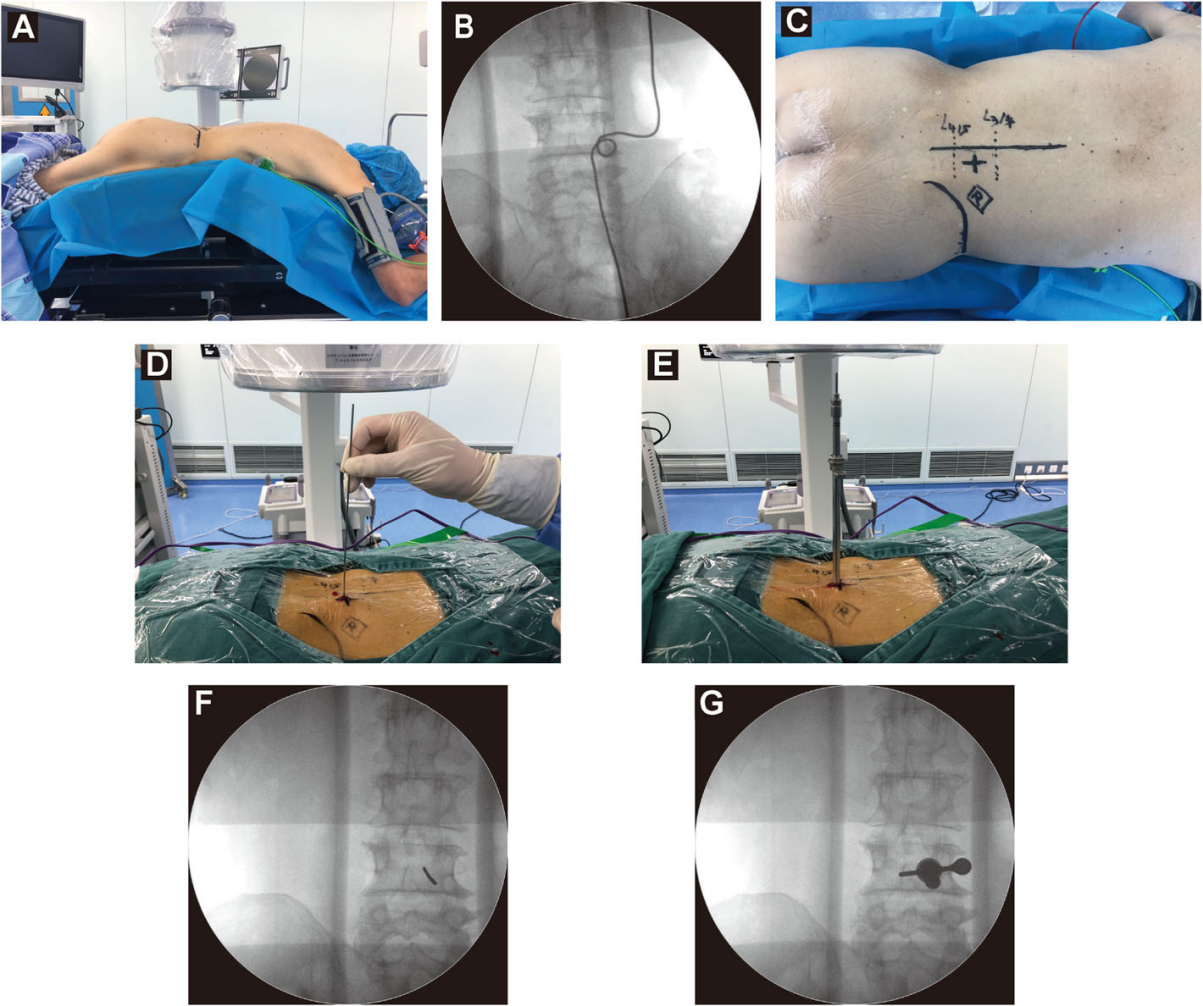
PELD combined with the VAT. **a** The patient was placed in the prone position with the hands naturally oriented at the sides of the head on a fluoroscopy-compatible arch frame. **b**, **c** The medial-inferior edge of the lamina was localized in the target segment and marked on the body surface. **d** A 2-mm Kirschner wire was vertically inserted into the target site of the lamina surface. **e** A trepan was used for further expansion of the working channel

The patient was placed in the prone position with the hands naturally oriented at the sides of the head on a fluoroscopy-compatible arch frame. The patient’s head was placed on a silicone doughnut headrest to ensure that he/she could breathe without difficulty and communicate with the surgeon during the procedure. The hip and knee joints were slightly flexed (Fig. 2a).

#### Localization and establishment of the working channel combined with the VAT

**Fig. 3:**
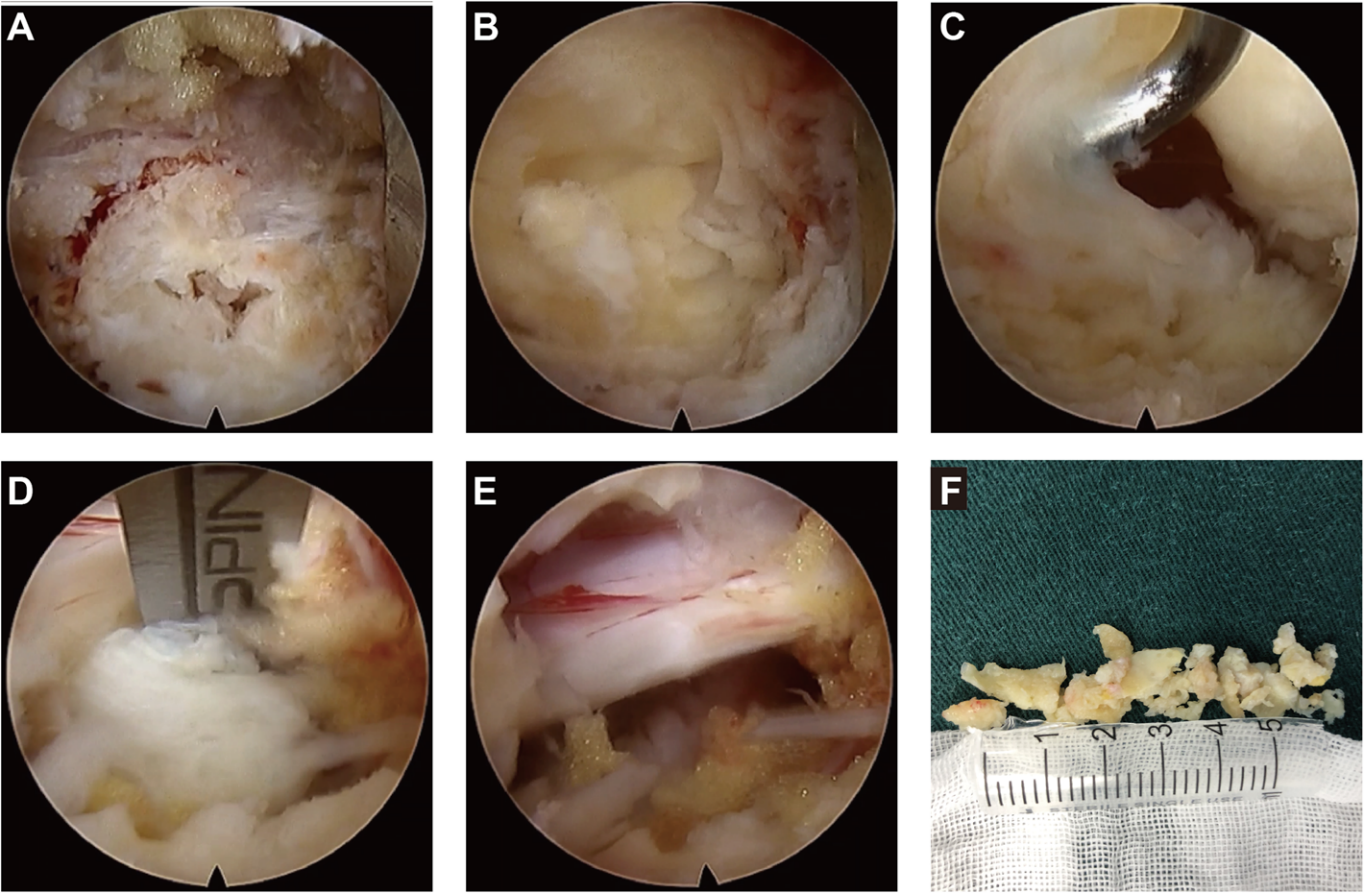
PELD combined with the VAT for LDH with distant upward migration, under the endoscope. **a** The “target” marker. **b** The bone tunnel. **c** A nerve hook was used to explore the ligamentum flavum and expand the gap between the ligamentum flavum and the dural sac. **d** A nerve stripper was used to explore the posterior nerve root to locate the herniated disc and release the tissue surrounding the nerve. **e** Complete removal of the surrounding tissue of the nerve root. **e, f** The specimen

After satisfactory positioning of the patient, fluoroscopy was performed using a C-arm X-ray machine to localize the medial-inferior edge of the lamina in the target segment to mark the distal projection point of the herniated disc on the body surface (Fig. 2b, c). Five millilitres of local anaesthetic solution (lidocaine:bupivacaine 2:1) was injected into the skin, soft tissue and periosteum. After satisfactory anaesthesia was achieved, a 0.8-cm incision was made at the marked skin site. To shorten the operative time, reduce intraoperative fluoroscopy times and increase the safety of the surgery, we used the VAT as follows: a 2-mm Kirschner wire was vertically inserted into the target site of the lamina surface. Fluoroscopy was performed using the C-arm X-ray machine to confirm the desired location of the Kirschner wire (Fig. 2f). A series of dilation tubes was used to expand the working channel. A trepan was used for further expansion of the working channel (Fig. 2e) and removal of the soft tissue along the working channel path. Fluoroscopy was repeated to confirm the establishment of a satisfactory working channel (Fig. 2g). Under an endoscope, the soft tissue around the target was removed, and a bipolar RF device was used for haemostasis. The “target” marker was clearly visualized under the endoscope, which helped the surgeon to find the target site accurately and quickly (Fig. 3a).

#### Establishment of a bone tunnel

A 3-mm high-speed grinding drill was used to establish a bone tunnel in the target made by the VAT. The diameter of the bone tunnel was 9 mm (three times the diameter of the drill bit). When the ligamentum flavum was visualized, a nucleus pulposus clamp and laminectomy rongeur were used to remove the superficial yellow ligament tissue until the ligament tissue became translucent, which indicated that the spinal canal was readily accessible. Then, a nerve hook was used to explore the ligamentum flavum and expand the gap between the ligamentum flavum and the dural sac. After confirming that no adhesions were present, the ligamentum flavum and the surrounding soft tissue could be completely removed. Then, the structure within the spinal canal was visualized (Fig. 3b, c). This step was executed carefully to reduce the risk of tearing the dural sac.

#### Removal of the disc tissue

After the spinal canal was completely exposed, the anatomical structures were carefully identified, and a nerve stripper was used to explore the posterior nerve root to locate the herniated disc and release the tissue surrounding the nerve, which prevented nerve damage or tearing of the dural sac when removing the disc. The nucleus pulposus clamp was used to remove the prominent nucleus pulposus of the intervertebral disc (Fig. 3d). Then, the distal end of the herniated disc was explored to assess whether residual disc tissue remained at the distal end due to breakage, and then exploration was continued down to the intervertebral disc level to remove the pedicle of the herniated disc. The bipolar RF device was used to ablate the rupture of the annulus of the intervertebral disc and to seal the rupture site to reduce the risk of herniation recurrence. After completely removing the herniated disc, exploration was repeated to confirm complete removal of the surrounding tissue. Normal pulsation of the nerve root with breathing was a sign to end the surgery (Fig. 3e). The postoperative specimen was routinely sent for pathological examination (Fig. 3f). The patient’s intraoperative condition, surgical tolerance, operative time and postoperative complications were recorded. Lumbar magnetic resonance imaging (MRI) was performed immediately after surgery to determine whether a residual nucleus pulposus remained and the effect of nerve root decompression.

### Postoperative management

Symptomatic treatment, including elimination of swelling, pain relief and nerve nutrition, was routinely applied after surgery. Twenty-four hours after bed rest, the patient was allowed to ambulate with waist protection. Three weeks after surgery, the patient was allowed to engage in normal activities with waist protection. A regular follow-up was performed. Postoperative improvement was assessed by comparing the preoperative and postoperative visual analogue scale (VAS), Oswestry Disability Index (ODI) and Japanese Orthopaedic Association (JOA) scores. The modified MacNab criteria were used to evaluate surgical efficacy.

### Statistical analysis

All statistical analyses were performed using SPSS version 19.0 statistical software (SPSS, Inc., Chicago, IL). Quantitative data are expressed as ( ±s). A *t* test was used to compare differences between 2 groups. *P* < 0.05 was considered statistically significant.

## Results

**Fig. 4:**
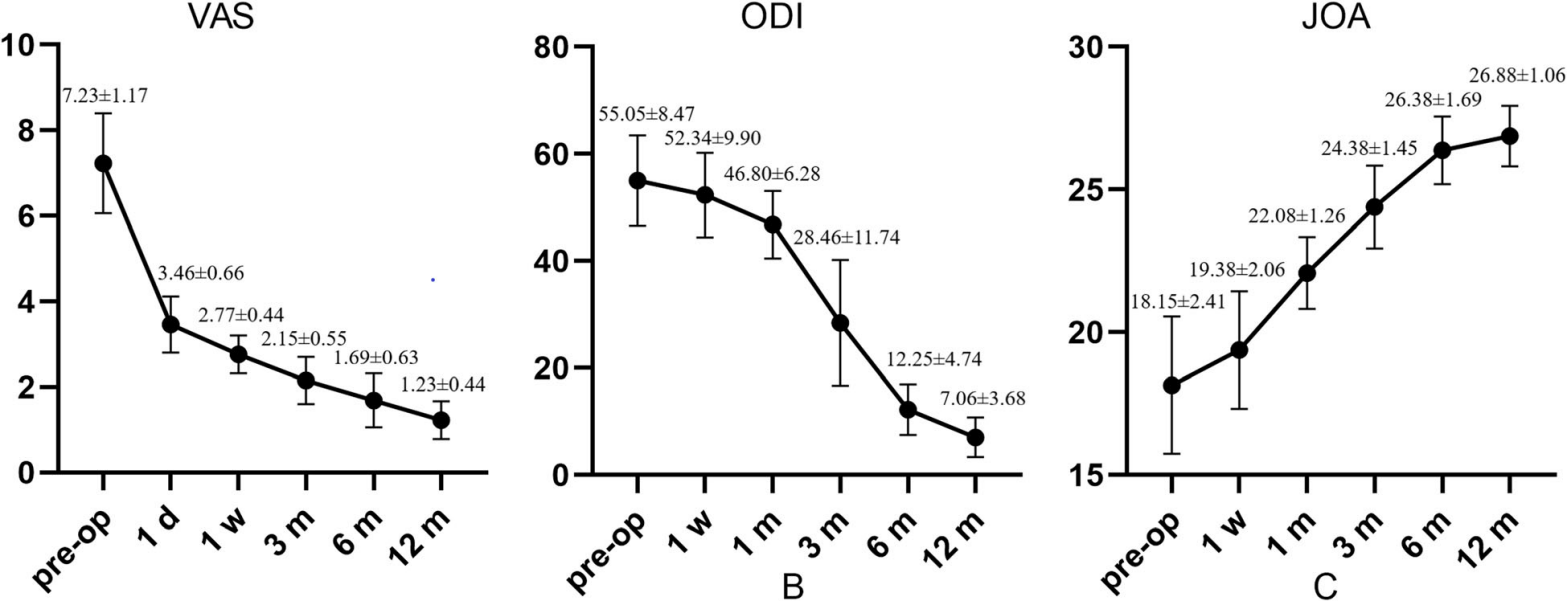
Preoperative and postoperative VAS, ODI and JOA scores (**a–c**)

**Fig. 5:**
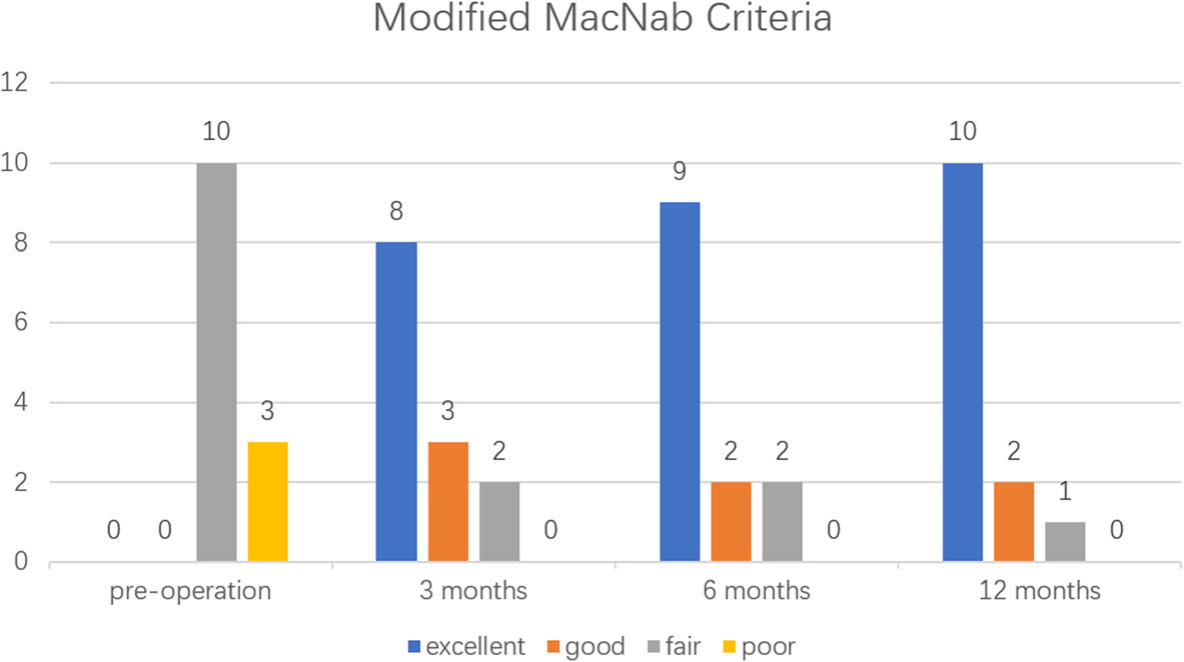
Clinical follow-up results according to the modified MacNab criteria

All 13 patients underwent successful surgery. The average operative time was 62.70 ± 19.11 min (range 40–110 min). The average hospital stay was 10.23 ± 4.46 days (range 4–17 days). Among the 13 patients, 1 experienced sensory disturbance after surgery and 1 had cerebrospinal fluid leakage, both of whom were cured after 1 month of conservative treatment (Table 2). No complications, such as nerve injury or wound infection, occurred. All 13 patients completed the follow-up visits for up to 12 months. The VAS was 7.23 ± 1.17 before surgery, 3.46 ± 0.66 at 1 day, 2.77 ± 0.44 at 1 week, 2.15 ± 0.55 at 3 months, 1.69 ± 0.63 at 6 months and 1.23 ± 0.44 at 12 months after surgery. The ODI values were 55.05 ± 8.47 before surgery, 52.34 ± 9.90 at 1 week, 46.80 ± 6.28 at 1 month, 28.46 ± 11.74 at 3 months, 12.25 ± 4.74 at 6 months and 7.06 ± 3.68 at 12 months after surgery. The mean preoperative JOA score was 18.15 ± 2.41, which increased to 19.38 ± 2.06 at 1 week, 22.08 ± 1.26 at 1 month, 24.38 ± 1.45 at 3 months, 26.38 ± 1.69 at 6 months and 26.88 ± 1.06 at 12 months after surgery. The differences in the scores before and after surgery were statistically significant (*P* < 0.05, Fig. 4). Twelve patients showed satisfactory effects according to the modified MacNab criteria by the last follow-up visit (Fig. 5).

**Table 2:** Operative characteristics

Operative time (mean) (range)	(62.70 ± 19.11) (40–110)
Hospital stay (mean) (range)	(10.23 ± 4.46) (4–17)
Complication	
Cerebrospinal fluid leakage	1
Sensory disturbance	1

## Discussion

The treatment of LDH has undergone tremendous changes in recent years [12, 13]. Since Kambin proposed PELD in 1973, this technique has gradually become well accepted after years of improvement and development. PELD has gradually become the first choice for the treatment of LDH, and a variety of surgical approaches are currently available [14–16]. LDH with distant upward migration was once considered a contraindication to PELD. Lee et al. concluded that LDH with downward migration can be treated with traditional PELD, but for distant upward migration, PELD was associated with a high risk of postoperative residual herniated disc tissue and damage to the stability of the facet joints. Thus, open surgery is a more secure option [4]. Choi et al. reached a similar conclusion by analysing 10,228 patients with LDH who underwent PELD. Choi’s study identified 436 failed cases, 283 of which were due to residual intervertebral disc tissue (64.9%). Among these cases, 70 were identified as migrated herniation (24.7%), and 11 cases were identified as distant migrated herniation (3.9%) [5]. Therefore, for the treatment of LDH with distant upward migration, complete removal of the lumbar disc tissue is the primary goal when selecting a surgical approach.

The transforaminal approach is used to remove the herniated disc at its base and is associated with an extremely high risk of residual herniated disc at the distal end. Furthermore, this approach for the removal of LDH with distant upward migration requires partial resection of articular processes and pedicles to achieve complete exposure, which undoubtedly disrupts the stability of the facet joint [4, 17]. In 2011, Kim et al. removed distant upward migrated herniated intervertebral discs through the contralateral intervertebral foramen approach. However, this method requires a longer working channel and is associated with a possible increased risk of injury [18]. In 2012, Dezawa et al. successfully removed intervertebral discs from a hidden zone by establishing a bone tunnel at the base of the pedicle in 9 patients but suggested that this procedure is difficult and not routine [19, 20]. With this surgical approach, no ligamentum flavum covers the medial region of the target, and removal of the bone structure can therefore allow direct access to the spinal canal, thus imposing a risk of damaging the dura mater during the process of establishing the bone tunnel. We selected a target site in the middle, medial and inferior margins of the lamina, and the medial margin was located at the insertion of the ligamentum flavum. After grinding to the level of the ligamentum flavum, a nucleus pulposus clamp, laminectomy rongeur and nerve hook can be used to safely and effectively access the spinal canal, thus minimizing the occurrence of iatrogenic injury. Correct selection of the target site substantially improves the safety of this approach.

**Fig. 6:**
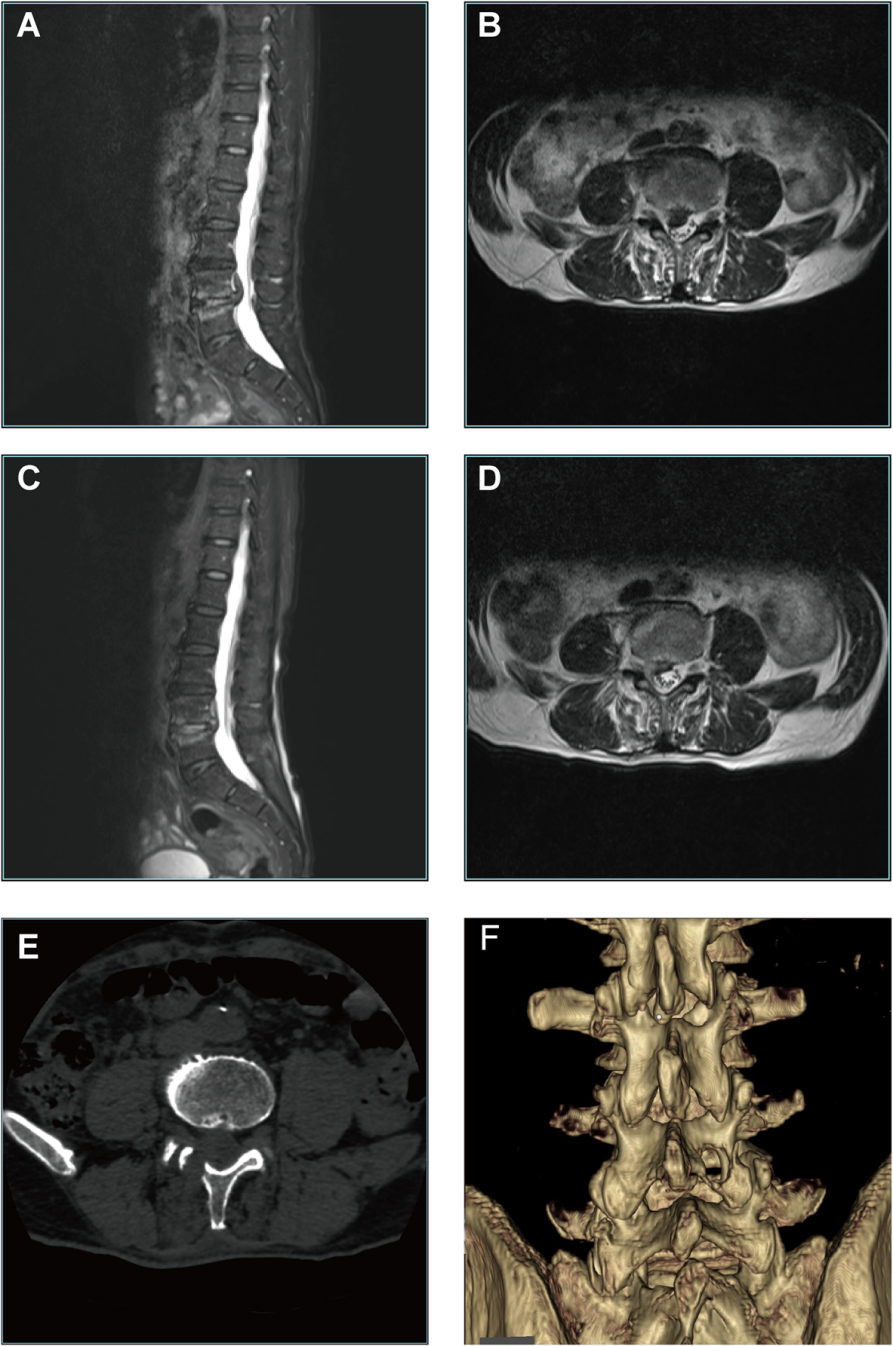
Case 1: a 74-year-old male patient was admitted to the hospital due to “pain and numbness in the right lower extremity for 19 h”. Lumbar MRI showed that the L4/5 intervertebral disc had migrated to zone 1 in the upper right part of the body (Fig. 6a, b). The patient underwent posterior PELD. During surgery, the herniated intervertebral disc was removed. MRI was repeated to confirm complete removal of the herniated disc in the spinal canal (Fig. 6c, d). Three-dimensional computed tomography (CT) reconstruction indicated that the bone tunnel was located at the lower edge of the lamina, which was consistent with the preoperative plan (Fig. 6e, f)

**Fig. 7:**
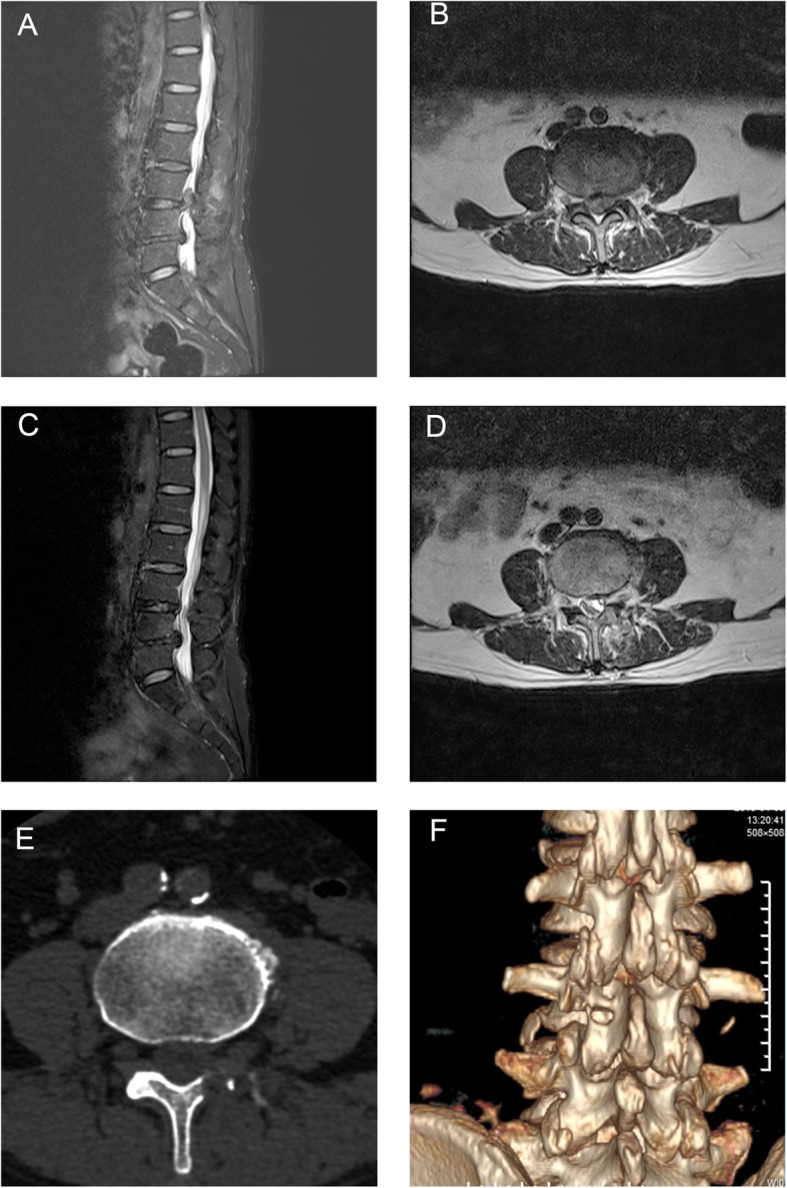
Case 2: a 62-year-old woman was admitted to the hospital because of “low back pain with pain in the medial aspect of the left lower leg for 2 months”. Lumbar MRI showed that the L3/4 disc had migrated to zone 1 in the posterior right upper side of the body, which was accompanied by L4/5 disc herniation (Fig. 7a, b). According to the patient’s signs and imaging examination, we determined that the patient’s symptoms were caused by L4 nerve root compression. Therefore, the patient underwent posterior PELD to remove the L3/4 upward migrated herniated disc. MRI was immediately repeated, which showed that the protruding tissue in the spinal canal was completely removed and that the spinal canal was re-expanded (Fig. 7c, d). Postoperative CT showed the bone tunnel (Fig. 7e, f)

How to locate the puncture target site accurately and quickly is the key to this technique. We applied the VAT for percutaneous endoscopic cervical discectomy (PECD) to PELD [21]. The VAT is a puncture technique that provides stable guidance for the establishment of the working channel by fixing a Kirschner wire. Surgeons can prevent deflection of the working sheath while establishing the working channel and identify the target area more easily under endoscopic view, which may significantly shorten the operation time. We used the C-arm X-ray machine to accurately identify the medial-inferior edge of the lamina. The Kirschner wire was inserted and anchored to the target site. During dilation of the channel, the trepan was used for second-stage expansion of the channel and made a circular mark on the lamina. Because the “target” created by the circular and the point mark made by the Kirschner wire can help us find the target site under the endoscope, we do not need a repeated X-ray to confirm the position of the working channel. Meanwhile, the soft tissue around the target can be removed quickly by the trepan, which can help surgeons identify the anatomic structure clearly and improve the efficiency of establishing the working channel. Application of the VAT greatly reduces the operative time and intraoperative fluoroscopy times, which can protect the surgeons and patients from radiation exposure. By establishing a bone tunnel in posterior PELD for the treatment of distant upward migrated LDH, surgeons can directly and accurately identify and remove the nucleus pulposus of the intervertebral disc from the distal end. Through the bone tunnel, exploration can continue down to the intervertebral disc level and up to the upper edge of the pedicle level to ensure complete removal of the intervertebral disc tissue and the mixed fragmented endplate tissue, which can substantially reduce the risk of postoperative residual herniated disc tissue in the spinal canal and improve the success rate of surgery. This procedure preserves the integrity of the facet joints while providing a clear view of the anatomical structures and significantly reduces the distance between the working channel and the protruding disc tissue and the risk of nerve damage. This surgical approach is not limited by the height of the crest of the ilium or the space between the lamina and can be used to treat distant upward migrated LDH at any segment. In addition, the procedure is performed completely under local anaesthesia. The patient can communicate with the doctor during the entire procedure, and the working position and operative target can be adjusted at any time, significantly improving the safety of the procedure (Figs. 6 and 7).

Of course, this investigation has certain limitations: (1) this was a retrospective study. All patients achieved good therapeutic effects without complications, such as nerve damage or wound infection, but this was a small-sized study. (2) The short follow-up limited our ability to observe the long-term efficacy of the procedure and complications. (3) This procedure can only be used as a supplement to the conventional surgical procedure and cannot be used as a routine surgical procedure for other types of LDH. Therefore, the application of this procedure must be based on careful preoperative planning and extensive experience in PELD.

## Conclusion

Posterior PELD combined with the VAT is a supplement to the PELD technique. This procedure has several advantages, including a shorter operative time, no damage to the facet joint and a lower risk of residual herniated disc tissue in the spinal canal. After a surgeon becomes proficient in PELD via the conventional approach, posterior PELD combined with the VAT can be a safe, effective and feasible surgical procedure for the treatment of LDH with distant upward migration.

## Data Availability

The datasets used and analysed during the current study are available from the corresponding author on reasonable request.

## Abbreviations

JOA: Japanese Orthopaedic Association
LDH: Lumbar disc herniation
ODI: Oswestry disability index
PECD: Percutaneous endoscopic cervical discectomy
PELD: Posterior percutaneous endoscopic lumbar discectomy
RF: Bipolar radiofrequency
VAS: Visual analogue score
VAT: Vertical anchoring technique
